# An Interpretation Architecture for Deep Learning Models with the Application of COVID-19 Diagnosis

**DOI:** 10.3390/e23020204

**Published:** 2021-02-07

**Authors:** Yuchai Wan, Hongen Zhou, Xun Zhang

**Affiliations:** Beijing Key Laboratory of Big Data Technology for Food Safety, School of Computer Science, Beijing Technology and Business University, Beijing 100048, China; 2030702057@st.btbu.edu.cn (H.Z.); zhangxun@btbu.edu.cn (X.Z.)

**Keywords:** visual interpretation, deep learning, machine learning, COVID-19, computer-aided diagnosis, CT images

## Abstract

The Coronavirus disease 2019 (COVID-19) has become one of the threats to the world. Computed tomography (CT) is an informative tool for the diagnosis of COVID-19 patients. Many deep learning approaches on CT images have been proposed and brought promising performance. However, due to the high complexity and non-transparency of deep models, the explanation of the diagnosis process is challenging, making it hard to evaluate whether such approaches are reliable. In this paper, we propose a visual interpretation architecture for the explanation of the deep learning models and apply the architecture in COVID-19 diagnosis. Our architecture designs a comprehensive interpretation about the deep model from different perspectives, including the training trends, diagnostic performance, learned features, feature extractors, the hidden layers, the support regions for diagnostic decision, and etc. With the interpretation architecture, researchers can make a comparison and explanation about the classification performance, gain insight into what the deep model learned from images, and obtain the supports for diagnostic decisions. Our deep model achieves the diagnostic result of 94.75%, 93.22%, 96.69%, 97.27%, and 91.88% in the criteria of accuracy, sensitivity, specificity, positive predictive value, and negative predictive value, which are 8.30%, 4.32%, 13.33%, 10.25%, and 6.19% higher than that of the compared traditional methods. The visualized features in 2-D and 3-D spaces provide the reasons for the superiority of our deep model. Our interpretation architecture would allow researchers to understand more about how and why deep models work, and can be used as interpretation solutions for any deep learning models based on convolutional neural network. It can also help deep learning methods to take a step forward in the clinical COVID-19 diagnosis field.

## 1. Introduction

Coronavirus disease 2019 (COVID-19) is an infectious disease that has infected more than 88 million confirmed cases all over the world and caused more than 1.9 million deaths [1] as of 10 January in 2021. The number of confirmed cases is still increasing in a rapid speed. In Dec. 2020, Priesemann et al. [2] made a call for a strong and coordinated European response to develop a common vision to guide the management of the pandemic and maintain low case numbers. According to the forecast of Perc et al. [3], the daily growth rates should be kept at least below 5% if we wish to see plateaus any time soon. Reverse transcription polymerase chain reaction (RT-PCR) test [4] and chest computed tomography (CT) are the two important tools for confirming COVID-19 patients. The RT-PCR can be used for diagnosis, but occasionally repeated RT-RCR need to be tested to gain more accurate result [5,6]. Besides, RT-PCR test is time-consuming, which takes 4–6 h to obtain the results. Chest CT can detect the characteristics, e.g., ground glass opacity (GGO) or bilateral patchy shadows, manifested in the COVID-19 infected lung [7,8]. Hence CT can be used for the timely detection of COVID-19 infection. Chest CT is more sensitive but less specific than RT-PCR [9,10]. To alleviate the burden of clinician in reading CT scans, some studies [11,12,13,14,15,16] have been conducted trying to develop methods to identify the infected patients automatically based on computer-aided diagnosis (CAD) strategy. Most of these studies employ deep learning methods, especially the convolutional neural network (CNN), in order to classify images of CT scans as infected or not. The widespread use of CNNs for image classification tasks is due to the fact that they have gained high accuracy in the fields of natural image recognition and object detection [17].

While the deep learning based COVID-19 diagnostic methods have shown promising results, they still suffer from one major limitation that the explanation of deep learning model is challenging. Due to the high complexity and high non-transparency of deep models, most of the state-of-the-art deep learning-based diagnostic methods lack the interpretation of the deep models and the diagnostic decisions. The deep model is an end-to-end model (always called as “black-box”), and the details of data flow inside it are invisible. Its lack of decomposability into intuitive and understandable components makes it hard to be interpreted. Thus, it is hard to explain why and how the diagnostic decision is made and whether the decision is trustable. Thus, there may exist cases where the model can gain perfect diagnostic performance, but the information inside the model used to support the decisions are irrelevant or incorrect. Such cases are quite unacceptable in the key fields, such as medical imaging and biology, where a high degree of reliability is required. The non-transparency of deep learning methods hinders the application of them in the clinical COVID-19 diagnosis field. Therefore, it is of vital importance to make an explanation of the deep learning model. However, to our best knowledge, very little work has been reported on the comprehensive interpretation of deep learning methods, especially for COVID-19 diagnosis.

### 1.1. Related Work

#### 1.1.1. Deep Learning Approaches for COVID-19 Analysis

In recent years, the deep learning methods arise and have been widely used in a wide range of machine learning tasks, such as image classification [18,19,20], image segmentation [21,22], natural language processing [23,24], and etc. Due to the good performance of deep learning methods, it has been utilized for resolving the problem of automatic COVID-19 analysis by many researchers. The state-of-the-art deep learning approaches for COVID-19 analysis can be grouped into three categories: classification, detection, and segmentation, which are detailed as follows.

COVID-19 classification methods try to classify the CT or X-ray scans into infected, uninfected, or make a severity assessment about different stages. Farooq et al. [25] presented a CNN framework (called as COVID-ResNet) for differentiating COVID-19 cases from other pneumonia cases. They utilized a three step technique to fine-tune a pre-trained ResNet-50 architecture in order to reduce the training time and achieve the accuracy of 96.2%. Hemdan et al. [26] presented a COVIDX-Net framework to diagnose COVID-19 in X-ray images, which includes seven different architectures of deep convolutional neural network models, such as VGG19 and MobileNet-v2. As shown in [26], the best performances were achieved by the VGG19 and DenseNet classifiers with the F1-scores of 89.0% and 91.0%. Jamshidi et al. [12] rendered a response to combat the virus through artificial intelligence (AI). They made a discussion about the potentials of deep learning approaches for diagnosis and treatment of COVID-19. Kang et al. [14] proposed to conduct the diagnosis of COVID-19 with a series of features extracted from CT images. The CT images were mapped into a latent space to encode complementary information from different types of features and revealed the underlying class distribution. The highest diagnostic accuracy was 89.4%, achieved by the fully connected neural network with handcrafted features. He et al. [27] proposed a Self-Trans approach to classify COVID-19 and non-COVID-19 CTs, which integrated contrastive self-supervised learning with transfer learning to learn feature representations for reducing the risk of overfitting. They achieved the diagnostic accuracy of 85.0%. Abdani et al. [28] proposed a lightweight deep learning model with 14 layers of CNN to identify the COVID-19 disease for various severity levels. The proposed model achieved the mean accuracy of 95.0% with the lowest standard deviation among the training folds accuracy. Masood et al. [29] employed deep learning model to classify COVID-19 and healthy chest X-ray images, which used pre-trained deep CNN models to extract deep features and then used SVM for classification of these features. This combination achieved the highest accuracy of 94.7%. Shi et al. [30] made a review of the COVID-19 classification approaches, which grouped the classification approaches into three categories: classification of COVID-19 from non-COVID-19, classification of COVID-19 from other pneumonia, and severity assessment of COVID-19.

The detection approaches of COVID-19 try to detect and recognize the regions of COVID-19 lesions from the CT or X-ray scans. Hu et al. [13] proposed a weakly supervised deep learning framework to detect COVID-19-infected regions automatically using chest CT data acquired from multiple centers and multiple scanners. Based on the detection results, they further achieved the diagnosis result for the COVID-19 patients, and the highest diagnostic accuracy was 90.6%. Guiot et al. [31] proposed an automated detection framework, which extracted radiomics features from volumetric chest CT exams and then conducted detection of COVID-19 based on these features. Alom et al. [32] proposed a COVID_MTNet to identify COVID-19 patients with multi-task deep learning methods, in which both X-ray and CT scan images were considered. The detection model showed around 85.0% testing accuracy in X-ray images and 99.0% accuracy in CT-images. Qian et al. [33] proposed a focal loss-based neural network ensemble model for accurate COVID-19 detection on class imbalance data. In this model, a flexible module was designed to ensemble several convolutional neural network models and fuse with a focal loss. They achieved the precision of 78.3% and F1-score of 81.7%.

The segmentation approaches of COVID-19 try to segment the CT or X-ray scans into different regions. It delineates the regions of interest (ROIs), e.g., lung, bronchopulmonary segments, and infected regions, for further assessment and quantification. Shan et al. [34] proposed a deep learning based segmentation system to automatically quantify the infection regions as well as the entire lung from chest CT scans. They evaluated the system by comparing the automatically segmented infection regions with the manually delineated ones on 300 chest CT scans of 300 COVID-19 patients. The average dice similarity coefficient showed 91.6% agreement between automatic and manual segmentations. Zhang et al. [35] proposed a conditional generative model to learn deep models for lung and COVID-19 infection segmentation from a single radiological image by resorting to synthesizing diverse radiological images. They achieved the average dice similarity coefficient score of 93.0% and average normalized surface dice score of 76.0%. Segmentation can also be used as the first step of various COVID-19 applications, such as diagnosis and quantification. For the diagnosis application, Li et al. [36] used U-Net for lung segmentation, and then classified COVID-19 from community-acquired pneumonia based on the segmentation results. The per-scan sensitivity and specificity for detecting COVID-19 in the independent test set was 90.0% and 96.0%. Jin et al. [37] used the CT slices segmented by a segmentation network as the input, and conducted fast COVID-19 diagnosis. For the quantification application, Huang et al. [38] used a deep learning model to segment the lung region and GGO to quantitatively evaluate the lung burden changes in patients with COVID-19. Cao et al. [39] used the voxel-level deep learning-based method for CT segmentation of pulmonary opacities for improving quantification of COVID-19.

Even though many of the above methods achieved promising performance, most of them lack the reliability discussion and explanation about the deep learning model, except for the work of Hu et al. [13], which visualized the deep features using class activation mapping.

#### 1.1.2. Interpretation of Deep Learning Models

Besides high accuracy, high reliability of the deep learning based diagnostic model is also required, because a mis-diagnosis in the medical field may lead to serious results. Therefore, it is essential to make an explanation of the diagnostic models. While the deep neural networks enable promising performance in multiple tasks, their lack of decomposability into intuitive and understandable components makes them hard to be interpreted [40]. The deep neural network, often referred to as a black-box model, is an end-to-end architecture. It means that given an input, the model can output the result of it. But the data flow and computing process inside the deep neural network is invisible or hard to understand.

In recent years, some studies have been proposed for the explanation of deep neural networks. The strategies of state-of-the-art methods can be grouped into two classes: opening the black-box or closing the black-box. Studies of the first strategy try to look into the black-box and analyze the hidden layers of the neural network. The parameters inside the neural network are analyzed and visualized, such as the learned features, gradients, filters, neurons, and etc. Zhou et al. [41] proposed the method of generating class activation maps (CAM) using global average pooling (GAP) in CNNs, and then evaluated the localization ability of CAM by visualizing the class-specific saliency map. A drawback of CAM is that it requires feature maps to directly precede soft-max layers. Thus when the neural network is used for visualization, the network architecture needs to be modified and the parameters need to be retrained. To address this problem, Selvaraju et al. [42] proposed the gradient-weighted CAM (Grad-CAM) method to combine feature maps using the gradient signal that does not require any modification in the network architecture. This allows the approach to be applied to any CNN-based architecture. Several medical image-analyzing methods employed the Grad-CAM method as the visualization tool. For example, Tiulpin et al. [43] proposed a method based on the deep Siamese CNN to automatically score knee osteoarthritis severity, and used the Grad-CAM to provide the attention maps to highlight the radiological features affecting the network decision. Panwar et al. [44] proposed a deep transfer learning algorithm which accelerated the detection of COVID-19 cases on X-ray and CT scans. Then the Grad-CAM was utilized to interpret the detection process.

The second strategy keeps the neural network as a black-box, and tries to design interpretable surrogate models to approximate the behavior of neural network as closely as possible. Ribeiro et al. [45] proposed the LIME method to explain the predictions of any classifier by learning an interpretable model locally around the prediction. The flexibility of the method is demonstrated by explaining different models for text (e.g., random forests) and image classification (e.g., neural networks). To explore which part of the original input image is important for the decision-making process of the neural network, Xu et al. [46] assigned a confidence value to each patch of the input image using the classification model trained by linear SVM. To identify the most relevant brain area for Alzheimer’s disease, Rieke et al. [47] occluded an area of the brain image, and then analyzed the difference of diagnostic performance between the un-occluded and occluded cases.

### 1.2. Our Work and Contributions

In this paper, we propose a comprehensive interpretation architecture for deep learning models, and apply it to COVID-19 diagnosis on CT images. For the diagnosis, we first construct the diagnostic model based on Alexnet neural network to classify the CT images into with or without COVID-19 lesions automatically. Then, we compare the diagnostic performance of our deep learning model with the traditional classification method which utilizes the handcrafted feature of local binary pattern (LBP) and support vector machine (SVM) classifier. The deep model achieves the diagnostic result of 94.75%, 93.22%, 96.69%, 97.27%, and 91.88% in the criteria of accuracy, sensitivity, specificity, positive predictive value, and negative predictive value, which are 8.30%, 4.32%, 13.33%, 10.25%, and 6.19% higher than that of the compared traditional methods. The visualized features explain the reasons for the superiority of deep model. Then, we further analyze the different aspects of the training and testing phase of the deep model, such as the feature patterns in the hidden layers, support regions for diagnostic decisions, and etc.

**Figure 1:**
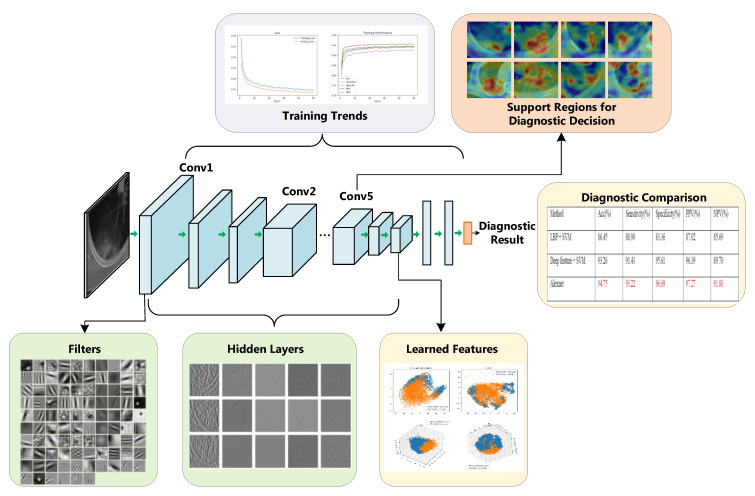
Framework of the proposed interpretation architecture for deep learning models. We interpret the deep model from different perspectives, including the training trends, diagnostic comparison, learned features, feature filters, neuron patterns in hidden layers, the support regions for diagnostic decision, and etc.

The framework of this paper is illustrated in Figure 1. In summary, the contributions of this paper are as follows.

(1) We propose an interpretation architecture of deep learning models, and apply the architecture to COVID-19 diagnosis. Our interpretation architecture has good generality and can act as the interpretation solution for any CNN-based deep learning classification methods in other research areas.
(2) Our architecture makes a comprehensive interpretation of deep models from different perspectives, including the training trends, diagnostic performances, learned features, feature extractors, neuron patterns in hidden layers, the areas of interest for supporting diagnostic decision, and etc. To our best knowledge, very little study has made a comprehensive interpretation about the deep learning-based diagnosis model. Furthermore, all of our interpretation results can be presented intuitively through visualization.
(3) Unlike most of the previous studies that focus on analyzing the testing phase of a CNN, in our architecture we propose to interpret the deep learning model from both the training phase and the testing phase to provide a general understanding of the CNN. The understanding of the training dynamic is quite necessary, through which researchers can observe the evolution of the diagnostic model, know whether the training is on the right track and whether the model converges, find latent mistakes, and etc.
(4) To analyze the diagnostic performance of the deep learning-based COVID-19 diagnostic model, we design the comparison between the deep learning model and traditional classification method. Furthermore, we novelly make an explanation about the difference of diagnosis accuracy through visualizing the learned features of both methods. To our best knowledge, very little study has made a performance comparison together with the visual explanation of the difference in performance.
(5) In our architecture, we provide a new possible pattern for CAD in the clinical field, through providing the supports for diagnostic decision. In the CAD of clinical COVID-19 diagnosis, our visualized attention maps for supporting diagnostic decisions can promisingly act as an important reference. For a new scanned CT image, our deep learning diagnostic model can output the diagnostic decision and the attention map for supporting the decision simultaneously. Clinician can first observe the diagnosis result, and then refer to the visualized attention map to make sure whether the diagnostic model focuses on the right regions, and whether the diagnosis result of the deep learning model for this image is trustable.
(6) Besides the interpretation architecture, we construct a deep learning model for the diagnosis of COVID-19. We choose the Alexnet framework as the deep learning model. Then we modify the original Alexnet to adapt it to the binary classification of COVID-19. The modified Alexnet model is trained using transfer learning strategy.
(7) Overall, our proposed interpretation architecture provides an intuitive analysis of the deep learning models, which can possibly help to build better trust and take a step forward in the application of CAD methods into the clinical COVID-19 diagnosis field. First, through the visualization of the training trends, the comparison and explanation of classification performance, and the visualization of the CNN layers, researchers can gain a general judgement about the robustness and accuracy of the deep model. Then, for each new CT image, the clinician can obtain the diagnostic result from the deep model, and then make sure whether the diagnostic result for this image is trustable by referring to the visualized attention maps as supports. The clinical COVID-19 diagnosis can benefit from the high efficiency and accuracy of the CAD systems, and obtain high reliability through our interpretation architecture at the same time.

## 2. Materials and the Diagnostic Model

### 2.1. Materials

To evaluate and interpret the diagnostic model, we collected the CT volumes of 285 patients and formed a dataset of 6233 CT images, with 3482 cases containing COVID-19 lesions and 2751 cases without COVID-19 lesions. The CT volumes were collected by Wuhan No.3 hospital, using Siemens CT scanners. The slice thicknesses of CT volumes were 3 mm, 5 mm, and 10 mm. The lesions in CT images were manually annotated by seven radiologists with 3–8 years of experience. The annotation results of each radiologist were confirmed by other radiologists to ensure the quality. We explored the patch-based method for diagnosis, where lesion patches cropped from the CT images were used as the input of the diagnosis framework. We trained and evaluated the diagnostic model using five-fold cross-validation, and the results were averaged to arrive at the final evaluation.

All the experiments were conducted on a computer with an Nvidia GeForce RTX 2080 Ti GPU and 256G memory.

### 2.2. The Deep Learning-Based Diagnostic Model

**Figure 2:**
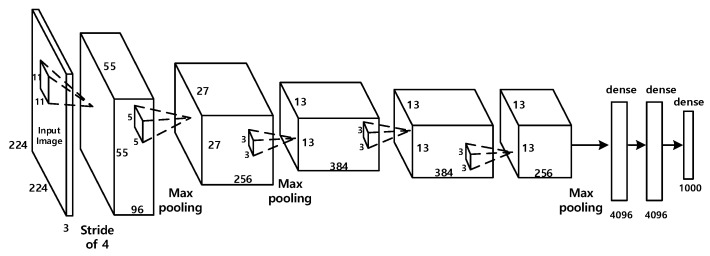
Structure of Alexnet. Alexnet contains eight learned layers with weights: five convolutional layers and three fully connected layers.

In this section, we design and construct the deep learning-based diagnostic model to classify the CT images into two classes: with or without COVID-19 lesions. We employ the widely used Alexnet [48] framework as the deep learning model for classification. The Alexnet is a typical CNN framework containing 60 million parameters and 650,000 neurons. As shown in Figure 2, it contains eight learned layers with weights: five convolutional layers and three fully connected layers. The Alexnet is originally designed for the task of natural image classification with 1000 classes. To adapt to the task of COVID-19 diagnosis, we modify the structure of Alexnet, and utilize the transfer-learning strategy to train the parameters of the network.

**Figure 3:**
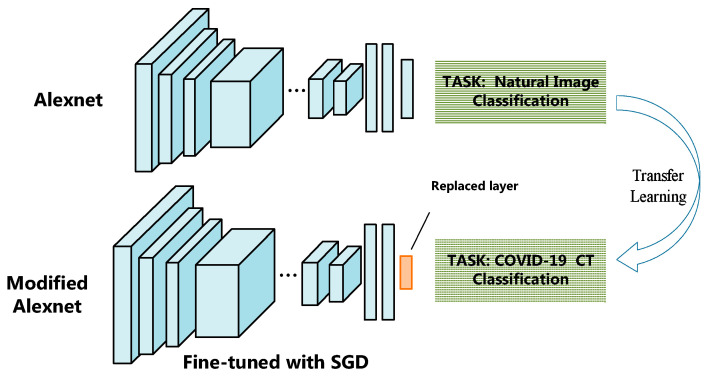
The modification and transfer learning of Alexnet. The last layer of AlexNet is replaced using our layer with two neurons to output the binary diagnostic result.

In the original Alexnet, the last layer contains 1000 neurons corresponding to the 1000 natural image classes. As our COVID-19 diagnosis work is a binary classification task, we modify the Alexnet to adapt to our task. We replace the last layer of AlexNet using our layer with two neurons to output the binary diagnostic result, as illustrated in Figure 3.

The training from scratch of the modified Alexnet requires large scale of image dataset, and is time consuming. We adopt the transfer learning strategy because of the lack of the prerequisite large scale of labeled images in medical image-analyzing field. Transfer learning is a very convenient and effective method to train deep neural network when there are not enough labeled samples [49]. In our work, we utilize the Alexnet pre-trained using the large scale data set of ImageNet [50]. Then the parameters in the pre-trained network are used as initiation, and the network is fine-tuned using the COVID-19 CT image dataset in our paper.

The modified AlexNet is trained by the algorithm of stochastic gradient descent (SGD) with momentum. In the training process, the hyper-parameters of learning rate, the batch size, and the number of iterations are set as 0.0001, 32, and 50, respectively.

## 3. The Interpretation Architecture and Results

In this section, we employ the modified Alexnet model to diagnose the COVID-19 CT images and interpret the diagnostic model from different perspectives. We first train the model parameters to obtain the optimal diagnostic model and visualize the training dynamic in the training phase. Then, the trained model is utilized to classify the CT images into two classes: with or without COVID-19 lesion. We then compare the classification performance and explain the reasons for the difference in performance. We further analyze the hidden layers in our CNN model to visualize what the diagnostic model has learned from the images. At last, we calculate and visualize the attention maps of the deep model to show the support regions for diagnostic decision.

### 3.1. Training Dynamic

Most of the previous studies investigate what features have been learned by a CNN. However, little research focuses on visualizing the training dynamic. Nevertheless, the understanding of the training process is quite necessary, through which researchers can know whether the training is on the right track, whether the model converges, find latent mistakes, and etc.

**Figure 4:**
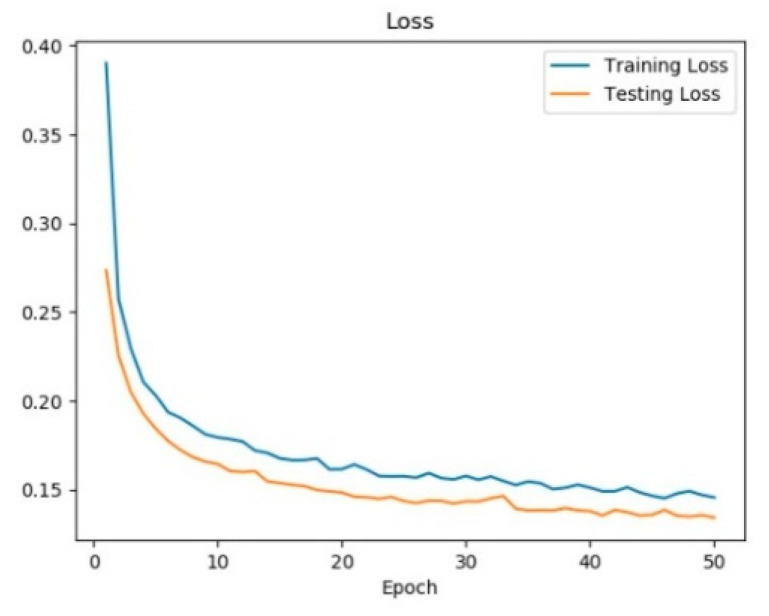
The loss on training and testing sets with the increase of epoch. At the beginning of the training phase, the model is relatively poor with high loss; then the loss decreases rapidly; then the model is improved gradually and at last it converges. Notice that the testing loss is lower than the training loss. The reason is that we used dropout strategy in the training phase, which was not included in the testing phase.

In this paper, we explore the analysis of training phase of our deep learning-based COVID-19 diagnostic model. For the training of the modified Alexnet, we divided the dataset as training set and testing set. We set the number of training epoch as 50, and recorded the loss in each epoch with the cross entropy loss function. The result is shown in Figure 4.

**Figure 5:**
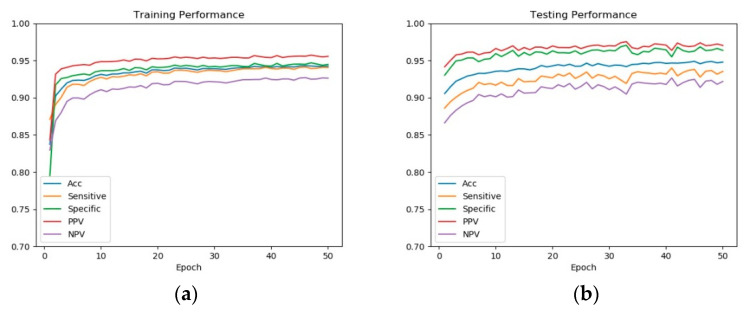
The classification performance with the increase of epoch: (**a**) results on training set, (**b**) results on testing set. At the beginning of the training phase, the model is relatively poor with low classification performance; then the performance increases rapidly; then the model is improved gradually and at last it converges.

Furthermore, we recorded the classification performances of the intermediate states of the model in each epoch. Five widely used criteria of classification performance were used: classification accuracy rate (*ACC*), sensitivity, specificity, positive predictive value (*PPV*), and negative predictive value (*NPV*). Let TP, TN, FP, and FN be the number of true positives, true negatives, false positives, and false negatives, respectively. Then the *ACC*, sensitivity, specificity, PPV, and NPV are measured as Equation (1) to Equation (5), respectively. The results on training set and testing set with the increase of epoch are shown in Figure 5.

(1) $$ACC=\frac{TP+TN}{TP+TN+FP+FN}$$

(2) $$Sensitivity=\frac{TP}{TP+FN}$$

(3) $$Specificity=\frac{TN}{TN+FP}$$

(4) $$PPV=\frac{TP}{TP+FP}$$

(5) $$NPV=\frac{TN}{TN+FN}$$

From Figure 4 and Figure 5, we can see that: at the beginning of the training phase, the model is relatively poor with high loss and low classification performance; then in the following epochs the model is improved at a rapid speed with loss decreases and performance increase rapidly; then the model is updated and improved gradually and at last it converges to the optimal model.

The visualization of the training phase provides a general idea of the training phase and the evolution of COVID-19 diagnostic model. The visualization result demonstrates that our model is trained in the right track.

### 3.2. Diagnostic Performance

To evaluate the diagnostic performance of our final trained deep learning model, we conducted the COVID-19 classification task. To make a comparison of the diagnostic performance as well as the image features, we chose the traditional classification method for comparison, which utilized handcrafted feature of LBP (59-dimensions) and SVM classifier. The classification results are presented in Table 1, with the best result in each criterion colored in red.

From Table 1, we can see that our modified Alexnet model achieves the best result in all the compared methods. The results of our model are 8.30%, 4.32%, 13.33%, 10.25%, and 6.19% higher than that of the traditional classification method in the five criteria of accuracy, sensitivity, specificity, PPV, and NPV, respectively. The deep learning-based model can obtain an obvious better performance mainly because it can extract comprehensive deep features from images. Therefore, to verify the performance of deep features, we extracted the deep features from the modified Alexnet and then applied SVM to classify these features. The results of “deep feature + SVM” method are shown in Table 1. From Table 1, we can see that after transferring the image features from LBP to deep features, the SVM classifier achieves an obvious improvement, the results of which are 6.81%, 2.53%, 12.25%, 9.37%, and 4.01% higher than that of the traditional method in these five criteria, respectively. More comparisons of the features are detailed in Section 3.3. Besides, the results of “deep feature + SVM” method are still 1.49%, 1.79%, 1.08%, 0.88%, and 2.18% lower than that of modified Alexnet, which demonstrates the good diagnostic performance of our deep learning model.

### 3.3. The Learned Features

The image features used for classification is crucial to COVID-19 diagnosis task. As the feature is the representation of an image, the quality of the features has a great influence on the classification performance, which is preliminary demonstrated in Table 1.

**Figure 6:**
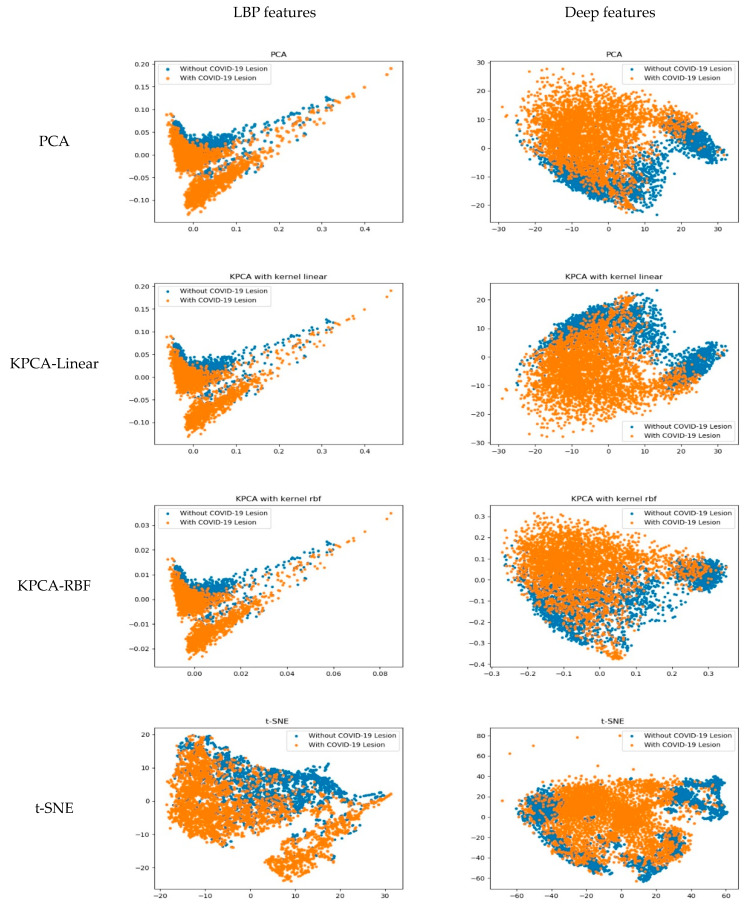
Visualization of local binary pattern (LBP) and deep features in 2-D space. The scatters in orange or blue colors denote the CT images with COVID-19 lesions or without COVID-19 lesions. We can see that the deep features are more discriminative than the LBP features.

**Figure 7:**
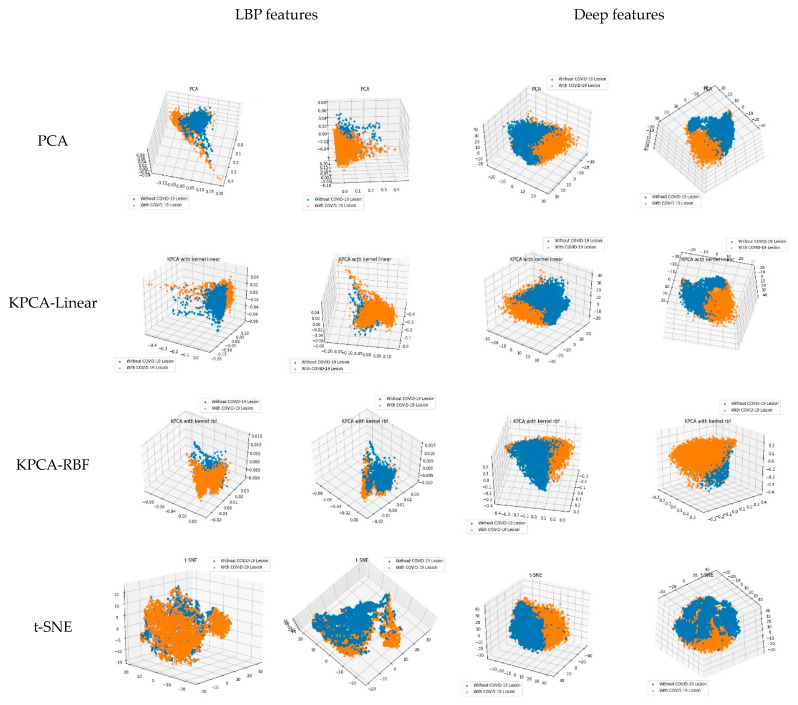
Visualization of LBP and deep features in 3-D space. For each method on each feature type, we picked two representative perspectives and displayed them.

In this section, we visualize and compare the learned features in the traditional machine learning method and the deep learning model. The deep model can learn feature representations from the input images. For each image, we extracted the output of the last layer of the feature extractor in Alexnet (as shown in Figure 1) to gain a feature vector of 9216-dimensions as the deep feature representation of it. The LBP features of each image were calculated using the traditional LBP operators. Then, we projected the feature vectors of LBP and deep features to 2-D or 3-D space using widely used dimensional reduction methods, namely the principal component analysis (PCA), kernel PCA (KPCA), and t-distributed stochastic neighbor embedding (t-SNE) methods. Finally, we displayed the images in our dataset in 2-D space and 3-D space, the results of which are shown in Figure 6 and Figure 7.

For the KPCA method, we employed two typical kernels in our experiments, namely the linear kernel and radial basis function (RBF) kernel. In Figure 6 and Figure 7, the four rows correspond to the results of PCA, KPCA-Linear, KPCA-RBF, and t-SNE, respectively. In each subgraph, the scatters in orange or blue colors denote the CT images with COVID-19 lesions or without COVID-19 lesions, respectively. From the 2-D visualization results in Figure 6, we can see that the deep features are more discriminative than the LBP features, which is demonstrated by the result of each dimensional reduction method. The scatters of LBP features are mixed together heavily, while that of the deep features are separable on the whole. This conclusion is confirmed by the 3-D results in Figure 7. In Figure 7, for the 3-D result of each method on each feature type, we picked two representative perspectives and displayed them. We can see that in the 3-D space, the deep features are more discriminative than the LBP features.

The visualization of learned features can make an explanation about the classification performance in Section 3.2. The deep model can extract more discriminative information about the images, thus the deep features provide better representation of the images.

### 3.4. Visualization of CNN Layers

A CNN is an end-to-end system, which takes in the images in the input layer and gives the result in the output layer. Between the input and output layers, many hidden layers exist. As it is hard for the CNN to be divided into intuitive parts, the explanation and understanding of the information inside CNN is difficult. The analysis and interpretation of the hidden layers inside CNN is essential for understanding the classification process and confirming the diagnosis result. In this section, we reveal and make an interpretation of the first layer filters and hidden layers of our deep learning model.

#### 3.4.1. First Layer Filters

A typical way of analyzing the features extracted by a first layer of a deep neural network is by looking at the “filters” learned by the network, which is the linear weights in the input-to-first layer weight matrix, represented in input space.

**Figure 8:**
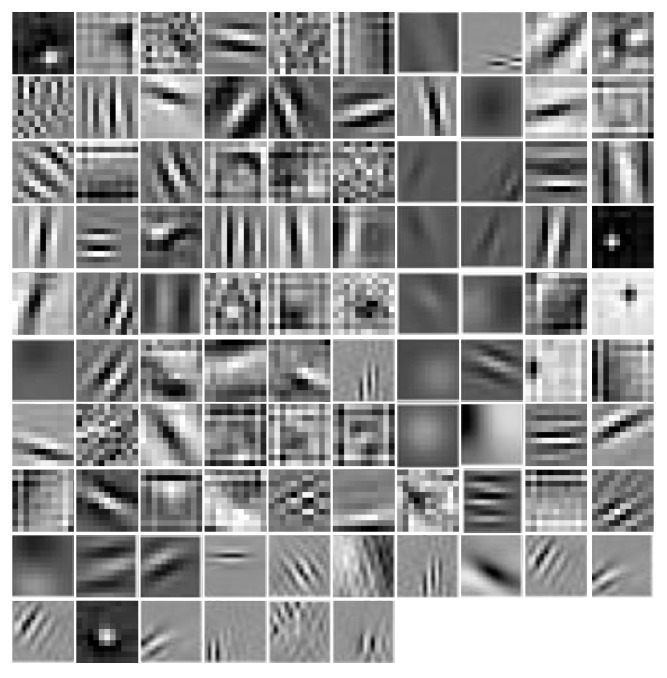
The visualization of first layer filters of modified Alexnet. There are 96 filters, each of which is in size of 11 × 11. The filters focus on the local pattern details of the images, and extract rich feature representations from the images.

In the first layer of Alexnet model, there are 96 filters, each of which is in size of 11 × 11. We mapped each filter of the first layer to an image and visualized the images in Figure 8. From Figure 8, the interesting finding reveals that the CNN attempts to imitate the human visual cortex system, in which the neurons in the lower layers are sensitive to basic patterns, such as shapes, margins, and lines [51]. We can observe that the first layer of modified Alexnet focuses on the local pattern details of the images, and can extract rich feature representations from images.

#### 3.4.2. Hidden Layers Visualization

Beyond the first layer, the neurons in the following layers gradually learn to extract features hierarchically. Compared with the first layer, understanding the hidden layers is more challenging.

To analyze the hidden layers of the modified Alexnet, we calculated and visualized the feature patterns learned by the neurons in hidden layers using guided backpropagation approach [52]. We guided the signal from a specific neuron and layer and then backward passed through the network to analyze the hidden image patterns in this neuron. A resulting image is reconstructed to show the part of the input image that most strongly activates this neuron. Let $F^{l}$ and $R^{l}$ denote the feature map and reconstructed image of layer l, the guided backpropagation method only passes the positive values of feature map and gradients backward, as shown in Equation (6). This strategy makes it suitable for finding the part of the image that maximizes the activation of a feature.

(6) $$R^{l}=\left(F^{l}>0\right)\cdot \left(R^{l+1}>0\right)\cdot R^{l+1}$$

**Figure 9:**
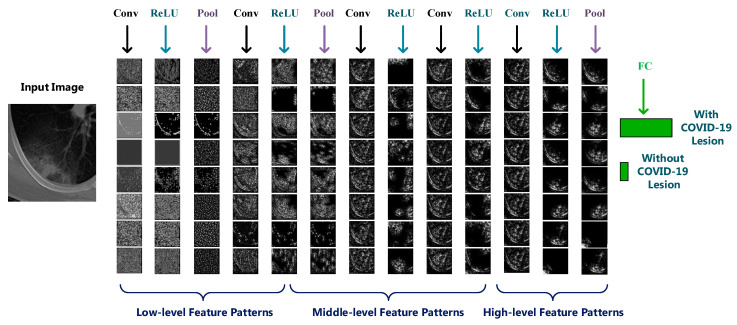
Visualization of layers in modified Alexnet for an input image. From the lower layers to higher layers, the deep model extract rich hierarchy of feature patterns from the input image, including low-level patterns, middle-level patterns and high-level patterns.

**Figure 10:**
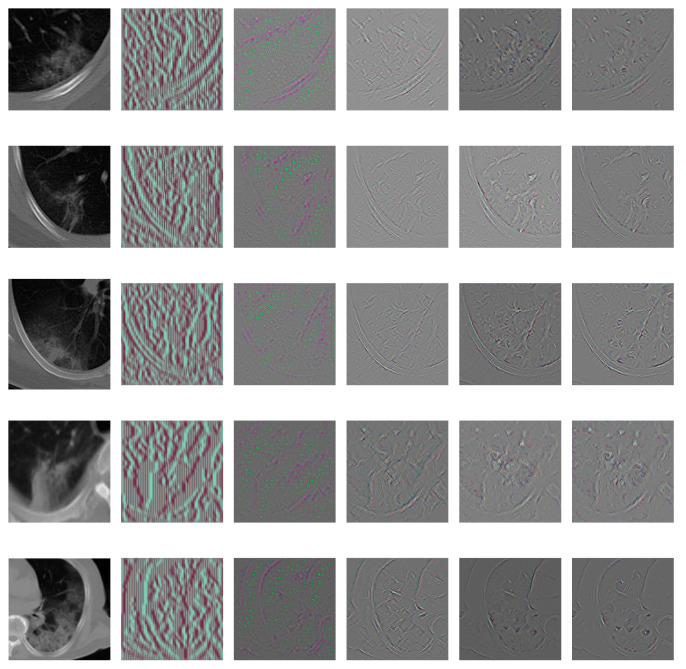

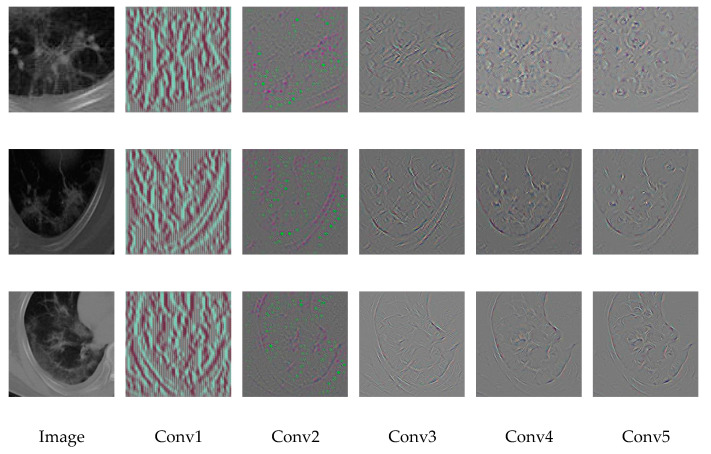
Visualization of hidden convolutional layers in modified Alexnet for eight example images. To clearly show the patterns, we generated the color images.

We visualized the learned patterns of the hidden layers [53] in Figure 9 and Figure 10. In Figure 9, we visualized the results of each layer of the modified Alexnet model on an example image infected with COVID-19. In each layer, eight neurons were randomly selected for visualization. In Figure 10, we visualized the results of the five hidden convolutional layers of Alexnet on eight example images infected with COVID-19. In each layer, one neuron was randomly selected for visualization. To clearly show the patterns, we generated the color images in Figure 10. In the experiments of Figure 9 and Figure 10, the example images were selected randomly.

From Figure 9 and Figure 10, we can observe that: (1) the multiple hidden layers can extract rich hierarchy of patterns from the input image, including low-level patterns, middle-level patterns, and high-level patterns. The lower layers extracted the detailed local patterns from images, such as the textures, margins, and etc. The complexity and variation of the visualized patterns increased when it comes to the middle layers. The higher layers extracted global pattern features, which include more semantic information. There is a pattern variation increment from lower layers to higher layers. (2) Different neurons interest in different information about the input image, which can be observed from Figure 9. Some neurons focus on the texture patterns, some extract the margins, some focus on the image local parts, and etc.

### 3.5. Support Regions for Diagnostic Decision

As mentioned above, in most previous studies, the CNN can make a classification about the input image, but the reasons for reaching this classification decision are not discussed. In this section, we analyze the areas of interest of our diagnostic model and visualize the attention maps for supporting the diagnostic decision.

The last convolutional layer of CNN contains rich semantic information about the input image, and plays an important role in making the final classification decision. We analyzed the feature maps and gradients of the last convolutional layer of the modified Alexnet, and generated class discriminative attention map using the Grad-cam method [41]. The heatmap of attention map displays the areas of interest of CNN for certain class in the decision-making process, which highlights image regions considered to be important for reaching the final diagnostic decision.

Let $y^{c}$ denote the outputted score of the network for class c. We first calculate the gradient of the score with respect to the *k*-th feature maps $A^{k}$ of the last convolutional layer, i.e., ∂yc∂Ak. Then, these gradients are global-average-pooled to obtain the weight $\alpha_{k}^{c}$ for feature map $A^{k}$ as:

(7) $$\alpha_{k}^{c}\;=\;\overset{global\;average\;pooling}{\overset{︷}{\frac{1}{R}\sum_{i}\sum_{j}}}\frac{\partial y^{c}}{\partial A_{ij}^{k}}$$

Then, we performed a weighted combination of the feature maps using Equation (8), and followed it by a ReLU to obtain the final attention map for class c using Equation (9).

(8) $$S^{c}=\sum_{k}\alpha_{k}^{c}A^{k}$$

(9) $$Map^{c}=ReLU(S^{c})$$

**Figure 11:**
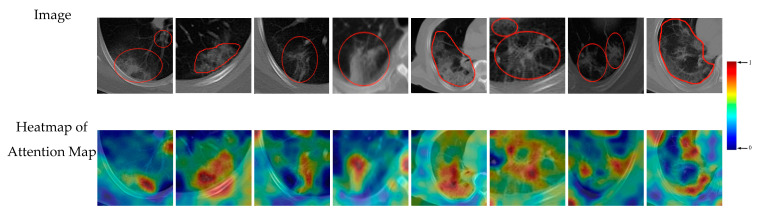
Heatmap of attention maps of modified Alexnet on eight example images. In most cases, the heatmaps can highlight the COVID-19 lesions automatically.

We generated the heatmaps of attention maps of eight example images, which were the same as the images of Figure 10. We visualized the results in Figure 11, where the heatmaps were overlaid onto the corresponding image to provide an intuitive view. From the visualization, we can see which area could affect the CNN classification decision. In Figure 11, regions with different colors denote different degrees of importance for the decision-making of CNN, where red color regions mean higher importance and blue color regions have lower importance. The first row in Figure 11 is the CT image with annotation, and the second row is the visualized result. From Figure 11, we can observe that in most cases the heatmaps can highlight the true relevant radiological findings (the COVID-19 lesions) automatically, which demonstrates the good localization ability of our deep learning models.

**Figure 12:**
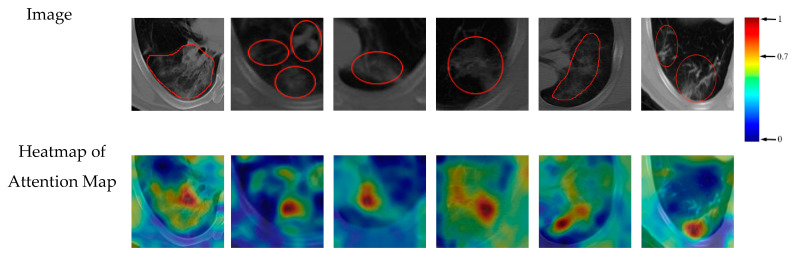
Heatmap of attention maps of the first six images with highest consistent degree.

From the visualization of the support regions, we can gain an intuitive observation of which area of the input image affects the classification decision. In the clinical application of CAD methods, the visualized attention maps may serve as important reference for clinicians. Referring to the attention maps, the clinician can judge whether the support regions of deep model are consistent to the lesion regions, and make a conclusion about the reliability of the diagnostic model. We further introduced the work of the clinicians to evaluate the consistent degree between the true lesion regions and the support regions provided by the deep model. 500 images from our dataset were selected for evaluation. For each image, the pixel with the heatmap value greater than 0.7 was considered as the support pixel, and the set of support pixels formed the support region. Then the overlapping area between the support region and the annotated lesion region was calculated. The proportion that the overlapping area takes in the lesion region was used as the consistent degree for this image. According to the results, the average consistent degree of 500 images is 60.02%. Furthermore, the first six images with the highest consistent degrees are visualized in Figure 12, with the heatmap value of 0.7 labeled in the colorbar.

## 4. Discussion

In this paper, we have proposed a visual interpretation architecture for explaining the deep learning models, and applied it to COVID-19 diagnosis. The comparison result of diagnostic performance demonstrated that our modified Alexnet deep model can achieve superior results than the traditional classification methods. The visualization result of the learned features illustrated that the deep features are more discriminative than the traditional handcrafted features, which explained the superiority of diagnostic performance. The visualized attention maps made it possible for the clinicians to observe the support regions for the diagnostic decisions of deep models. The calculated result of consistent degree between the true lesion regions and the support regions from the deep model demonstrated the good localization ability of our deep learning model.

However, it is worth to point out that this study still has limitations. First, we only made a classification about with or without COVID-19 lesions, but did not make a severity assessment (light, normal, or heavy) of COVID-19 cases. Second, the interactivity is not included in the interpretation architecture. If the interpretation architecture can interact with users, more specific interpretation results can be provided to users.

In the future work, we will introduce more interpretation strategies into our interpretation architecture. For example, we will design the visualization of the topological structure of network to help researchers understand the network better. In the topological structure, as there are millions of links between two layers of the deep model, we will use the geometry-based edge bundling method to cluster the links and provide a better visualization. We will also try to design an interpretable surrogate model based on decision tree to approximate the behavior of neural network. Using the decision tree, we can analyze the classification process of deep neural network in a visible way and explore the reasons for misclassification. Furthermore, we will build an interactive interpretation system, which can interact with users and provide specific visualization result as the request of users.

## 5. Conclusions

In conclusion, aiming at improving the transparency and reliability of the deep learning models, we have proposed an interpretation architecture to make a comprehensive analysis and visualization of these models. Our deep learning-based diagnostic model achieves the diagnostic result of 94.75%, 93.22%, 96.69%, 97.27%, and 91.88% in the criteria of accuracy, sensitivity, specificity, PPV, and NPV, respectively. Our interpretation architecture of the model could allow researchers to gain an insight into the deep models and help to build better trust for deep learning methods in the clinical COVID-19 diagnosis field.

## Figures and Tables

**Table 1:** Performance comparison for COVID-19 classification of different methods, with the best result in each criterion colored in red. Our modified Alexnet model achieves the best result in all the compared methods.

Method	ACC (%)	Sensitivity (%)	Specificity (%)	PPV (%)	NPV (%)
LBP + SVM	86.45	88.90	83.36	87.02	85.69
Deep feature + SVM	93.26	91.43	95.61	96.39	89.70
Modified Alexnet	94.75	93.22	96.69	97.27	91.88
